# The genetic and clinical characteristics of WFS1 related diabetes in Chinese early onset type 2 diabetes

**DOI:** 10.1038/s41598-023-36334-7

**Published:** 2023-06-05

**Authors:** Yating Li, Siqian Gong, Meng Li, Xiaoling Cai, Wei Liu, Simin Zhang, Yumin Ma, Yingying Luo, Lingli Zhou, Xiuying Zhang, Xiuting Huang, Xueying Gao, Mengdie Hu, Yufeng Li, Qian Ren, Yanai Wang, Xianghai Zhou, Xueyao Han, Linong Ji

**Affiliations:** 1 Departments of Endocrinology and Metabolism, Peking University People’s Hospital, Peking University Diabetes Center, No 11, Xizhimen South Street, Beijing, 100044 China; 2 Beijing Pinggu Hospital, No·59, Xinping North Street, Beijing, 101200 China

**Keywords:** Mutation, Diabetes

## Abstract

Diabetes is one of the most common phenotypes of Wolfram syndrome owing to the presence of the variants of the *WFS1* gene and is often misdiagnosed as other types of diabetes. We aimed to explore the prevalence of WFS1-related diabetes (WFS1-DM) and its clinical characteristics in a Chinese population with early-onset type 2 diabetes (EOD). We sequenced all exons of the *WFS1* gene in 690 patients with EOD (age at diagnosis ≤ 40 years) for rare variants. Pathogenicity was defined according to the standards and guidelines of the American College of Medical Genetics and Genomics. We identified 33 rare variants predicted to be deleterious in 39 patients. The fasting [1.57(1.06–2.22) ng/ml] and postprandial C-peptide levels [2.8(1.75–4.46) ng/ml] of the patients with such *WFS1* variations were lower than those of the patients without *WFS1* variation [2.09(1.43–3.05) and 4.29(2.76–6.07) respectively, ng/ml]. Six (0.9%) patients carried pathogenic or likely pathogenic variants; they met the diagnostic criteria for WFS1-DM according to the latest guidelines, but typical phenotypes of Wolfram syndrome were seldom observed. They were diagnosed at an earlier age and usually presented with an absence of obesity, impaired beta cell function, and the need for insulin treatment. WFS1-DM is usually mistakenly diagnosed as type 2 diabetes, and genetic testing is helpful for individualized treatment.

## Introduction

Wolfram syndrome (WS) is a rare neurodegenerative disease characterized by diabetes mellitus (DM), optic atrophy (OA), deafness (D), diabetes insipidus (DI), and other psychiatric disorders. The approximate prevalence rate of WS was reported to be 1 in 54,478 in the Sicilian district of Italy and 1 in 770,000 in the United Kingdom^1–3^. The prevalence of WS is relatively high in some populations. In early studies, the prevalence of WS in the overall diabetic population was approximately 0.57% and 4.8% in the Lebanese population^1,4^.

Two classes of WS (WS1 and WS2) are known, which are attributed to variants of the *WFS1* gene or *CISD2* gene.WS1 is the most common type of WS, accounting for ~ 90% of all patients with WS. The prevalence of WS1 varies in different types of diabetes; a few studies with small sample sizes showed approximately 1.3% in type 1 diabetes mellitus, 0.025% in type 2 diabetes mellitus, 0.074% in early onset diabetes (EOD), and 3.5–4.3% in monogenic diabetes^5–7^, suggesting that WS1 is usually misdiagnosed as other types of diabetes.

The *WFS1* gene was mapped to the region 4p16.1 and comprises eight exons. Exon 1 is non-coding, exons 2–7 are small coding exons, and exon 8 is the largest exon. *WFS1* encodes the 890 amino acid long glycoprotein wolframin, which is composed of nine transmembrane segments and is localized primarily in the endoplasmic reticulum (ER) membrane. Wolframin is an important regulator of ER homeostasis. Despite being ubiquitously expressed, differences between tissues with high (i.e. pancreatic β-cells and brain) and with low (i.e. whole blood or kidney) expression are quite significant. Variants of *WFS1* are responsible for high levels of ER stress, activation of the unfolded protein response (UPR), effects on insulin processing and secretion, and induction of apoptosis in neurons and islet beta cells^8^.

To date, over 200 different WFS1 variants have been identified in patients with WS1, including missense, nonsense, and frameshift insertion or deletion variants, and even large deletions^9^. Most of these are located in exon 8, which encodes the transmembrane and C-terminal domains of the protein. WS1 is usually inherited in an autosomal recessive pattern, while dominant WFS1 variants were observed to associate with mild manifestations that appeared in an isolated manner, such as adult-onset diabetes, optic atrophy, congenital cataracts, and hearing impairment^10–14^.

The criteria for WS and WS-like disorders have been well described in the clinical guidelines for the management of WS (proposed by EURO-WABB, http://euro-wabb.org/guidelines/guidelines/). The minimal criteria for the diagnosis of WS are juvenile-onset diabetes mellitus and optic atrophy, and only approximately half of the patients with WS have the full phenotype associated with DM, OA, D, and DI. Some variants show partial features and are associated with poorly defined Wolfram Syndrome-Like disorders (WSLD), including diabetes mellitus, optic atrophy, and deafness in the dominant or recessive model. Therefore, here, we favor the use of the term “WFS1-related diabetes” (WFS1-DM) to describe the diabetes phenotype including WS and WSLD.

As recently reported, some adolescent patients with WS-like disorders present only with isolated diabetes^4,5,10^ and are usually misdiagnosed with type 1 or type 2 diabetes. As some types of antidiabetic drugs, such as glucagon-like peptide 1 receptor agonists (GLP-1RAs) and pioglitazone, might alleviate ER stress in islet beta cells and delay the development of diabetes^15,16^, a cost-effective clinical screening strategy and subsequent correct diagnosis based on genetic testing are necessary for timely initiation of individualized treatment before the onset of severely impaired beta cell function. However, the prevalence and clinical features of the patients with WFS1-DM have not been thoroughly investigated in the population with EOD. Thus, in the present study, we screened variants across exons of WFS1 in patients with clinically diagnosed EOD, described the genetic and clinical features of patients with WFS1-DM, and examined methods to clinically identify patients with WFS1-DM.

## Materials and methods

### Participants

A total of 690 unrelated patients with EOD were included in our cohort. Their clinical features are shown in Table 1. Clinical information was collected at the time of enrollment from the inpatient or outpatient department in the Department of Endocrinology and Metabolism at Peking University People’s Hospital from April 2013 to June 2019. All patients were of northern Han Chinese ancestry and were Beijing residents. They were diagnosed with diabetes mellitus in accordance with the 1999 World Health Organization (WHO) criteria. The inclusion criteria were as follows: (i) diagnosis of type 2 diabetes before the age of 40 years; (ii) demonstration of neither classical clinical presentation (i.e., complete lack of endogenous insulin secretion) of type 1 diabetes nor positive islet cell antibody (ICA) and glutamic acid decarboxylase antibody (GADA); (iii) absence of mitochondrial A3243G mutation; and (iv) absence of diseases of the exocrine pancreas and drug. According to Chinese-specific criteria, the patients were diagnosed with obesity if their BMI was ≥ 28 kg/m^2^ and overweight if their BMI was ≥ 24 kg/m^2^. Informed consent was obtained from the patients or their parents/guardians using a protocol approved by the Ethics Committee of Peking University People’s Hospital, in accordance with the Declaration of Helsinki.

**Table 1:** Clinical characteristics of patients with early-onset type 2 diabetes.

	EOD (n = 690)
Sex; male/female	473/217
Duration of diabetes; years	3.0 (0.1–8.0)
Age; years	34.0 (29.0–39.0)
Diagnosis age; years	30.0 (26.0–33.0)
Smoking history; n(%)	246 (35.7)
Family history; n(%)	466 (67.5)
OHA; n(%)	431 (62.5)
Insulin therapy; n(%)	258 (37.4)
Lipid-lowering drug; n(%)	54 (7.8)
BMI; kg/m^2^	27.8 ± 4.9
Waist, cm	
Male	97.0 ± 12.4
Female	90.5 ± 13.0
SBP; mmHg	127.0 ± 17.3
DBP; mmHg	80.0 (73.0–88.0)
ALT; U/L	26.0 (16.0–45.0)
AST; U/L	21.0 (16.0–30.0)
BUN; mmol/L	4.56 (3.69–5.59)
Scr; μmol/L	61.0 (51.0–73.0)
UA; μmol/L	
Male	377.0 ± 104.4
Female	319.5 ± 105.5
TC; mmol/L	4.81 (4.16–5.56)
TG; mmol/L	1.79 (1.22–2.90)
LDL-c; mmol/L	2.93 ± 0.91
HDL-c; mmol/L	0.99 (0.87–1.14)
hs-CRP; mg/L	1.81 (0.87–3.84)
CK; U/L	78.0 (57.0–113.0)
eGFR; ml/min/1.73m^2^	133.37 (116.14–156.72)
FPG; mmol/L	8.15 (6.34–10.75)
PPG; mmol/L	12.93 (10.29–15.90)
FCP; ng/ml	2.05 (1.41–2.95)
PCP; ng/ml	4.17 (2.68–6.06)
Fins; μU/ml	12.27 (7.56–19.80)
Pins; μU/ml	40.55 (24.70–68.79)
HbA1c, %	9.2 ± 2.4
HbA1c, mmol/mol	77.0 ± 2.7
UACR; mg/g	10.65 (4.49–39.93)
Obesity; n(%)	325 (47.1)
CHD; n(%)	22 (3.2)
Stroke; n(%)	21 (3.0)
Peripheral atherosclerosis; n(%)	193 (28.0)
Hypertension; n(%)	151 (21.9)
Diabetic nephropathy; n(%)	127 (18.4)
Diabetic retinopathy; n(%)	90 (13.0)

Statistical analysis was performed using SPSS 23.0. Normally distributed continuous variables are presented as the means and standard deviations (± SD), and non-normally distributed variables are presented as medians (25–75th percentile). Categorical variables are presented as numbers and percentages.

*EOD* early-onset type 2 diabetes, *OHA* oral hypoglycemic agents, *BMI* body mass index, *WHR* waist-hip ratio, *SBP* systolic pressure, *DBP* diastolic pressure, *ALT* serum glutamic pyruvic transaminase, *AST* serum glutamic oxalacetic transaminase, *BUN* blood urea nitrogen, *Scr* serum creatinine, *UA* uric acid, *TC* total cholesterol, *TG* triglyceride, *LDL-c* low-density lipoprotein cholesterol, *HDL-c* high-density lipoprotein cholesterol, *CK* serum creatine kinase, *hs-CRP* high sensitivity C-reactive protein, *eGFR* estimate glomerular filtration rate, *FPG* fasting plasma glucose, *PPG* 2-h plasma glucose during 75 g oral glucose tolerance test (OGTT), *FCP* fasting plasma C-peptide, *PCP* postprandial serum C-peptide, *Fins* fasting serum insulin, *Pins* postprandial serum insulin, *HbA1c* hemoglobin A1c, *UACR* urinary albumin/creatinine ratio, *CHD* coronary atherosclerotic heart disease.

### Clinical examinations and laboratory tests

Blood samples were collected in the morning after the patients had fasted for 10–12 h. Laboratory tests were conducted as described previously^17^. The estimated glomerular filtration rate (eGFR) was calculated using the following formula: eGFR, ml/min/1.73 m^2^ = 175 × Serum creatine (Scr, mg/dl) − 1.234 × age (years) − 0.179 × 0.79 (if female)] The serum GADA (RSR Limited, UK), ICA (Biomerica, USA) were tested using commercialized ELISA kit. Each subject underwent an ophthalmic examination using a TRC NW100 fundus camera (Topcon Corporation, Tokyo, Japan) or an ophthalmoscope. The following definitions were used: macrovascular complications included coronary atherosclerotic heart disease (CHD), peripheral vascular atherosclerosis (PA), and stroke. Microvascular complications include diabetic nephropathy (DN) and retinopathy (DR). CHD was diagnosed according to medical history (presence of angina or acute myocardial infarction and the results of computed tomography or coronary angiography). Cerebrovascular disease was defined as a history of transient ischemic attack or ischemic or hemorrhagic stroke. PA was diagnosed on the basis of carotid or lower-extremity arterial ultrasonography findings. DN was diagnosed if the urinary albumin/creatinine ratio was ≥ 30 mg/g, and/or an eGFR was < 60 ml/min/1.73 m^2^. DR and OA were diagnosed based on the results of a fundus camera or direct ophthalmoscopy. Hypertension was characterized by the use of antihypertensive drugs or with a systolic blood pressure ≥ 140 mmHg and/or diastolic blood pressure ≥ 90 mmHg.

### DNA sequencing

Whole blood samples were collected to extract genomic DNA. Next-generation sequencing (NGS) was performed to screen all exons of WFS1. Whole-exome or target sequencing was performed using the Roche NimbleGen Human Exon V2 capture chip or customized Agilent chip on the Illumina Hiseq2500 system or Hiseq4000 platform, respectively. The annotation tools were used that integrated several population databases, including the 1000 Genomes Project and Exome Aggregation Consortium database (ExAC), and Genome Aggregation Database (gnomAD) and disease phenotype databases, such as ClinVar and the National Center for Biotechnology Information (NCBI) Reference Sequence Database.

The variants were selected according to the following processes: First, the non-silent variants (non-synonymous, frameshift, in-frame indels, and splice-site variants) were selected. Secondly, variants with a frequency of less than 0.005 in the following population databases were included: ExAC (East Asian) and the 1000 Genome Project (Chinese). Finally, all the included variants were validated by Sanger sequencing.

### Bioinformatics analysis and evaluation of pathogenicity of WFS1 variants

The pathogenicity of these variants was assessed using five pathogenicity prediction algorithms (PROVEAN and SIFT, http://provean.jcvi.org; PolyPhen2, http://genetics.org). bwh.harvard.edu/pph2; MutationTaster, http://www.mutationtaster.org; MutationAssessor, http://mutationassessor.org). If variants were predicted to be deleterious using at least one algorithm, they were defined as deleterious. The pathogenicity of the variants was determined according to the standards and guidelines recommended by the American College of Medical Genetics and Genomics (ACMG).

WS or WSLD was diagnosed according to the aforementioned guidelines (EURO-WABB). If the patient met two major criteria (DM < 16 years or OA < 16 years) or one major criterion and two minor criteria [DI or DM > 16 years or OA > 16 years or sensorineural deafness or neurological signs (ataxia, epilepsy, cognitive impairment), renal tract abnormalities (structural or functional), or one pathogenic/likely pathogenic variant of WFS1 and/or family history of WS)] or carried two pathogenic/likely pathogenic WFS1 variants, he (she) was diagnosed. If a patient met one of the criteria for DM, OA, or deafness and carried at least one pathogenic/likely pathogenic WFS1 variant, he (she) was diagnosed with WSLD.

### Statistical analysis

The Social Science software package (SPSS for Windows, version 23.0, Chicago, Illinois, USA) was used for statistical analysis. The normally distributed continuous variables are expressed as mean and standard deviation (± SD) and the non-normally distributed variables as the medians (25–75th percentile). Categorical variables were expressed as numbers and percentages. Student t-test was used to test the mean of the quantitative traits, and the X^2^ test or Fisher's exact test was used for qualitative characteristics comparisons. The Mann–Whitney U test was used for non-normally distributed variables. Linear regression analysis was used to analyze the correlation between variables and confounding factors. Statistical significance was set at *P* < 0.05 was considered significant.

### Ethics statement

The study protocol was approved by the Ethics Committee of Peking University People’s Hospital (China), the Approval Number is 2014-06 and 2017PHB035-01.

## Results

### Clinical characteristics of study participants

The characteristics of the patients with EOD have been summarized in Table 1. Their mean age was 34.0 (29.0–39.0) years, the mean age at diagnosis was 30.0 (26.0–33.0) years, and their average BMI was 27.8 ± 4.9 kg/m^2^. Of these, 67.5% had a family history of DM, and 47.1% were obese. In terms of diabetic complications, 18.40% and 13.0% of the patients had DN and DR, respectively, and the prevalence of CHD, stroke, and hypertension was 3.2%, 3.0%, and 21.9%, respectively.

### DNA sequencing results

All rare variants identified in 690 diabetic patients in the cohort are shown in Table 2. We identified 44 individuals who carried 38 rare variants, including 5 variants (H67L, V503I, A616S, F783L, and S855L) that were predicted to be non-deleterious by all five programs in 5 patients and 33 variants that were predicted to be deleterious by at least one program in 39 patients.

**Table 2:** Rare coding variations (n = 38) of WFS1 in patients with early-onset type 2 diabetes.

Position	Region	Codon change	AA change	ACMG*	Allele frequency	
					1000 Genomes	ExAC
6,303,126	Exon8	TGGTGTGCTTCATGT-T	L535Lfs*3	P	0	0
6,303,589	Exon8	CTGCAGCCACC-C	L689Lfs*18	P	0	0
6,303,602	Exon8	GA-G	E694Gfs*16	P	0	0
6,290,799	Exon4	GCG-GTG	A134V	LP	0	0
6,293,030	Exon5	CAAG-C	K193del	LP	0	0
6,303,407	Exon8	CGG-TGG	R629W	LP	0	0
6,279,355	Exon2	GCG-GTG	A58V	US	0	0.0007
6,279,382	Exon2	CAT-CTT	H67L	US	0	0
6,290,847	Exon4	GCG-GTG	A150V	US	0.005	0.00124
6,292,998	Exon5	GCA-ACA	A179T	US	0	0.00008
6,293,076	Exon5	GGC-AGC	G205S	US	0	0.00012
6,296,873	Exon7	GAG-GCG	E273A	US	0	0.00032
6,302,413	Exon8	ATG-ATA	M297I	US	0	0
6,302,626	Exon8	AGC-AGG	S368R	US	0	0
6,302,741	Exon8	CAT-TAT	H407Y	US	0	0
6,302,756	Exon8	GTC-CTC	V412L	US	0.002	0
6,302,816	Exon8	CTG-GTG	L432V	US	0	0
6,302,927	Exon8	TCG-ACG	S469T	US	0	0
6,302,937	Exon8	CCC-CTC	P472L	US	0	0
6,303,029	Exon8	GTC-ATC	V503I	US	0.002	0.00016
6,303,030	Exon8	GTC-GGC	V503G	US	0	0.00016
6,303,060	Exon8	TAT-TGT	Y513C	US	0	0
6,303,062	Exon8	CTC-TTC	L514F	US	0	0
6,303,197	Exon8	GCC-ACC	A559T	US	0	0.00223
6,303,203	Exon8	ATC-GTC	I561V	US	0	0.00012
6,303,246	Exon8	GCC-GGC	A575G	US	0	0
6,303,368	Exon8	GCC-TCC	A616S	US	0.001	0.00004
6,303,378	Exon8	TCT-TTT	S619F	US	0	0
6,303,479	Exon8	CGC-TGC	R653C	US	0	0.00078
6,303,491	Exon8	ATG-TTG	M657L	US	0	0
6,303,575	Exon8	CGC-TGC	R685C	US	0	0.00036
6,303,644	Exon8	CGC-TGC	R708C	US	0	0.00008
6,303,869	Exon8	TTC-CTC	F783L	US	0	0.00028
6,303,891	Exon8	TCG-TTG	S790L	US	0	0
6,303,936	Exon8	CGG-CAG	R805Q	US	0	0.00008
6,304,086	Exon8	TCA-TTA	S855L	US	0	0.00008
6,304,097	Exon8	CGG-TGG	R859W	US	0	0.00016
6,304,134	Exon8	GTG-GGG	V871G	US	0	0.00044

RefSeq: NP_005996.2, *AA change* amino acid change, *1000 Genomes* 1000 Genomes Project-East Asian, *ExAC* exome aggregation consortium database-East Asian.

*The classification of rare coding variants according to the guideline of the American College of Medical Genetics and Genomics. Evidences of pathogenicity was detailed in Supplementary Table 1. *P* pathogenic, *LP* likely pathogenic, *US* uncertain significance.

The rare variants included missense mutations (89.5%), small deletions (7.9%), and in-frame deletions (2.6%). Most variants (78.9%) were located in exon 8, which encodes the putative transmembrane and COOH-terminal domains of wolframin. Two, two, three, and one variants were located in exon 2, 4, 5, and 7, respectively, all of which encode the NH_2_-terminal cytosolic domains of the protein (Supplementary Fig. 1). Eight variants (H67L, M297I, S469T, P472L, L535Lfs*3, S619F, L689Lfs*18 and E694Gfs*16) were identified (Supplementary Fig. 1 and Table 2). This finding may be in line with those of several previous functional studies examining the pathogenicity of three variants (A134V, K193del and R629W)^18–20^^.^ Among all the rare variants, according to ACMG, three variants (E694Gfs*16, L535Lfs*3, and L689Lfs*18) were pathogenic, three variants (A134V, K193del, and R629W) were likely pathogenic, and the others were uncertain (Table 2).

In addition to the 36 patients with heterozygous rare variants, three patients carried compound heterozygous rare variants that were all predicted to be deleterious. According to the ACMG, two patients carried compound heterozygous rare variants of uncertain significance (V412L and I561V, G205S, and A559T), and one patient carried compound heterozygous rare variants of uncertain significance (R685C) and a pathogenic variant (L535Lfs*3).

In summary, we identified six patients with WFS1-DM, and the prevalence of WFS1-DM was 0.9% in the Chinese population with EOD. Of the six patients with WFS1-DM, five carried only one heterozygous pathogenic/likely pathogenic variant and one carried two rare variants (one pathogenic variant and one variant of uncertain significance) (Table 4).

### Clinical features of patients with rare and predicted deleterious variants of WFS1

The clinical characteristics of patients with rare and predicted deleterious (at least one program) variants of WFS1 are summarized in Table 3. Their FCP and postprandial serum C-peptide (PCP) levels were lower than the patients without WFS1 rare variation [1.57(1.06–2.22) vs 2.09(1.43–3.05) ng/ml, *P* = 0.007 (*P* = 0.027, adjustment of BMI); 2.80(1.75–4.46) vs 4.29(2.76–6.07) ng/ml, *P* = 0.027 (*P* = 0.034, adjustment of BMI)]. The serum glutamic oxaloacetic transaminase (AST) levels of patients with rare variants of WFS1 that were predicted to be deleterious variants were higher than those without WFS1 rare variants [25.6(19.5–36.5) vs. 21.0(16.0–30.0) U/L, *P* = 0.025].

**Table 3:** Clinical characteristics of patients with rare and predicted to be deleterious variants and without rare variants in WFS1.

	Patients with rare and predicted to be deleterious variation*	Patients without rare variation	*P*
n	39	646	–
Sex; male/female	30/9	439/207	0.289
Duration of diabetes; years	3.0 (0.1–10.5)	2.0 (0.1–8.0)	0.675
Age; years	34.0(28.5–40.5)	34.0 (29.0–39.0)	0.909
Diagnosis age; years	30.0 (25.5–32.0)	30.0 (26.0–33.0)	0.268
Smoking history; n(%)	15 (37.8)	227 (35.1)	0.736
Family history; n(%)	35 (88.6)	501 (77.5)	0.143
OHA; n(%)	22 (56.3)	406 (62.8)	0.461
Insulin therapy; n(%)	19 (48.7)	236 (36.6)	0.098
Lipid-lowering drug; n(%)	6 (15.3)	48 (7.4)	0.175
BMI; kg/m^2^	27.1 ± 5.0	27.8 ± 4.9	0.190
Waist, cm			
Male	97.0 ± 12.4	97.0 ± 12.4	0.980
Female	84.8 ± 14.6	90.8 ± 12.8	0.173
SBP; mmHg	126.0 (116.0–140.0)	125.0 (116.0–138.0)	0.987
DBP; mmHg	80.0 (70.0–87.0)	80.0 (73.0–88.0)	0.991
ALT; U/L	32.0 (19.0–59.3)	26.0 (16.0–45.0)	0.135
AST; U/L	25.6 (19.5–36.5)	21.0 (16.0–30.0)	0.025
BUN; mmol/L	4.72 (3.82–5.67)	4.56 (3.70–5.54)	0.508
Scr; μmol/L	67.0 (56.0–76.0)	61.0 (50.0–72.0)	0.021
UA; μmol/L			
Male	407.4 ± 126.1	374.3 ± 102.1	0.146
Female	291.6 ± 59.0	320.9 ± 107.3	0.393
TC; mmol/L	4.63 (3.98–5.21)	4.82 (4.18–5.57)	0.259
TG; mmol/L	1.50 (1.15–1.96)	1.82 (1.23–2.97)	0.083
LDL-c; mmol/L	2.79 ± 1.07	2.94 ± 0.90	0.347
HDL-c; mmol/L	0.98 (0.85–1.13)	0.99 (0.87–1.15)	0.874
Male	0.97 ± 0.26	0.98 ± 0.22	0.738
Female	1.20 ± 0.21	1.12 ± 0.31	0.452
hs-CRP; mg/L	1.89 (0.78–3.23)	1.81 (0.88–3.86)	0.767
CK; U/L	82.80(55.0–135.0)	78.0 (57.0–111.0)	0.260
eGFR; ml/min/1.73m^2^	123.38 (113.54–143.91)	133.53 (116.32–157.19)	0.060
FPG; mmol/L	8.02 (6.89–11.96)	8.17 (6.34–10.70)	0.627
PPG; mmol/L	13.58 (11.01–16.27)	12.90 (10.20–15.88)	0.559
FCP; ng/ml	1.57 (1.06–2.22)	2.09 (1.43–3.05)	0.007(0.027)^#^
PCP; ng/ml	2.80 (1.75–4.46)	4.29 (2.76–6.07)	0.027(0.034)^#^
Fins; μU/ml	9.84 (6.28–14.64)	12.24 (7.56–18.54)	0.211
Pins; μU/ml	34.95 (22.40–48.36)	39.98 (23.18–64.20)	0.461
HbA1c; %	9.1 (7.1–11.2)	9.0 (7.3–10.9)	0.830
HbA1c, mmol/mol	76 (54–99)	75 (56–96)	0.830
UACR, mg/g	8.95 (4.80–29.96)	11.00 (4.47–42.08)	0.669
Obesity; n(%)	15 (39.50)	308 (47.7)	0.404
CHD; n(%)	1 (2.6)	20 (3.1)	1.000
Stroke; n(%)	2 (5.3)	19 (3.0)	0.433
Hypertension; n(%)	8 (21.1)	143 (22.1)	1.000
PA; n(%)	16 (41.4)	176 (27.2)	0.098
Diabetic nephropathy; n(%)	7 (18.4)	120 (18.5)	1.000
Diabetic retinopathy; n(%)	5 (13.2)	84 (13.0)	0.979

Statistical analysis was performed using SPSS 23.0. Normally distributed continuous variables are presented as the means and standard deviations (± SD), and non-normally distributed variables are presented as medians (25–75th percentile). Categorical variables are presented as numbers and percentages.

*Five patients with rare variants of WFS1 gene that the software predicted to be benign were excluded.

^#^*P* values in brackets were based on the results of linear regression analysis after adjusting for BMI.

*EOD* early-onset type 2 diabetes, *OHA* oral hypoglycemic agents, *BMI* body mass index, *WHR* waist-hip ratio, *SBP* systolic pressure, *DBP* diastolic pressure, *ALT* serum glutamic pyruvic transaminase, *AST* serum glutamic oxalacetic transaminase, *BUN* blood urea nitrogen, *Scr* serum creatinine, *UA* uric acid, *TC *total cholesterol, *TG* triglyceride, *LDL-c* low-density lipoprotein cholesterol, *HDL-c* high-density lipoprotein cholesterol, *CK* serum creatine kinase, *hs-CRP* high sensitivity C-reactive protein, *eGFR* estimate glomerular filtration rate, *FPG* fasting plasma glucose, *PPG* 2-h plasma glucose during 75 g oral glucose tolerance test (OGTT), *FCP* fasting plasma C-peptide, *PCP* postprandial serum C-peptide, *Fins* fasting serum insulin, *Pins* postprandial serum insulin, *HbA1c* hemoglobin A1c, *UACR* urinary albumin/creatinine ratio, *CHD* coronary atherosclerotic heart disease, *PA* peripheral atherosclerosis.

We identified six patients with WFS1-DM; their features and genetic analysis results are shown in Table 4 and Supplementary Fig. 2, respectively. All patients were diagnosed with diabetes before 35 years of age, and 50% (3/6) were younger than 25 years of age. None of the patients were obese, whereas two patients were overweight. Almost none of the patients had features associated with insulin resistance, such as obesity, hypertension, or hyperuricemia. Ketosis and microvascular and macrovascular complications were also rare in patients with WFS1-DM, except in one patient (Patient 2) with DN. Additionally, Patient 2 had a diabetes family history of three generations, and combined with her diagnosed age, she was clinically suspected of maturity-onset diabetes of the young (MODY). Patient 5 also presented with deafness. At recruitment, Patient 6 was taking oral hypoglycemic agents but had poor glucose control. Four patients used insulin, while 3 them were beyond the HbA1c target (< 7%).

**Table 4:** Clinical features of patients with WFS1-DM.

	Patient 1	Patient 2	Patient 3	Patient 4	Patient 5^#^	Patient 6
Clinical diagnosis	WSLD	WSLD	WSLD	WSLD	WSLD	WSLD
Variants	R685C/L535Lfs*3	L689Lfs*18	E694Gfs*16	K193del	A134V	R629W
Pathogenesis*	US/P	P	P	LP	LP	LP
Sex	Male	Female	Male	Male	Female	Male
Duration; years	44	0.1	5	0.1	9	0.1
Age; years	60	24	29	35	39	34
Diagnosis age; years	16	24	24	35	30	34
Smoking history	–	N	N	N	N	N
Family history	N	Y	Y	Y	Y	Y
Ketosis	–	N	N	N	N	N
BMI; kg/m^2^	–	17.3	27.2	24.7	21.8	20.9
Waist; cm	–	66	100	79	78	76
WHR	–	0.84	1.00	0.83	0.96	0.85
SBP; mmHg	–	110	120	120	129	100
DBP; mmHg	–	80	70	85	85	60
OHA	N	N	N	N	Y	Y
Insulin therapy	Y	N	Y	Y	Y	N
Lipid-lowering drug	–	N	N	N	N	N
ALT; U/L	25	6	73	688	24	23
AST; U/L	26	12	30	226	16	23
BUN; mmol/L	–	2.62	4.65	–	6.0	6.1
Scr; μmol/L	85.75	38	63	65.4	53	61
UA; μmol/L	315	192	371	259	260	350
TC; mmol/L	4.47	5.21	5.21	4.09	3.70	5.15
TG; mmol/L	0.85	1.23	1.44	1.48	1.24	3.09
LDL-c; mmol/L	2.49	3.51	3.68	2.14	1.98	3.51
HDL-c; mmol/L	1.79	1.14	1.00	1.50	1.20	0.95
hs-CRP; mg/L	1.44	4.82	3.18	0.75	0.92	0.17
CK; U/L	–	44	77	–	53	39
eGFR; ml/min/1.73m^2^	–	221.86	145.48	134.32	134.91	147.14
FPG; mmol/L	–	8.11	14.69	7.80	6.97	7.81
FCP; ng/ml	0.28	1.55	2.75	–	1.57	0.64
Fins; μU/ml	–	6.00	–	5.07	3.92	7.92
Pins; μU/ml	–	–	–	–	–	30.78
HbA1c; %	5.3	12.8	11.2	7.1	13.1	14.2
HbA1c, mmol/mol	34	116	99	54	121	132
UACR; mg/g	6.99	31.00	21.82	7.87	9.65	11.68
CHD	–	N	N	N	N	N
Stroke	–	N	N	N	N	N
PA	–	N	N	N	N	N
Hypertension	–	N	N	N	N	N
Hyperuricemia	–	N	N	N	N	N
Diabetic nephropathy	–	Y	N	N	N	N
Diabetic retinopathy	–	N	N	N	N	N

*N* no, *Y* yes,–: not available, *OHA* oral hypoglycemic agents, *BMI* body mass index, *WHR* waist-hip ratio, *SBP* systolic pressure, *DBP* diastolic pressure, *ALT* serum glutamic pyruvic transaminase, *AST* serum glutamic oxalacetic transaminase, *BUN* blood urea nitrogen, *Scr* serum creatinine, *UA* uric acid, *TC* total cholesterol, *TG* triglyceride, *LDL-c* low-density lipoprotein cholesterol, *HDL-c* high-density lipoprotein cholesterol, *CK* serum creatine kinase, *hs-CRP* high sensitivity C-reactive protein, *eGFR* estimate glomerular filtration rate, *FPG* fasting plasma glucose, *FCP* fasting plasma C-peptide, *Fins* fasting serum insulin, *Pins* postprandial serum insulin, *HbA1c* hemoglobin A1c, *UACR* urinary albumin/creatinine ratio, *CHD* coronary atherosclerotic heart disease, *PA* peripheral atherosclerosis.

*Pathogenesis of variants were determined according to ACMG: *P* pathogenic, *LP* likely pathogenic, *US* uncertain significance.

^#^Patient 5 was accompanied with hearing loss.

## Discussion

In the current study, we describe the prevalence, genetics, and clinical characteristics of WFS1-DM in a Chinese population. With a prevalence of 0.9%, WFS1-DM is common in the Chinese population with EOD. To the best of our knowledge, this is the first study of WFS1-DM in a Chinese population with EOD. The patients with WFS1-DM identified in our study were non-obese, diagnosed at an earlier age, had impaired beta cell function and required insulin use, and lacked ketosis and typical WS phenotypes. Serum C-peptide levels in carriers with rare variants and those with predicted deleterious variants were lower than those in the EOD group without rare variants. The AST levels in carriers of rare variants and those predicted to be deleterious variants were higher than those without WFS1 rare variants.

The differences in the prevalence of WFS1-DM noted among the studies can be attributed to the characteristics of the studied populations. No previous reports examining the prevalence of WFS1-DM in EOD in China have been published earlier, and several Chinese study cohorts were based on patients who had already been diagnosed with WS or carried WFS1 variants^21,22^. In our study, 6 patients with WFS-DM (0.9%) were identified among 690 Chinese patients with clinically diagnosed EOD. This percentage is higher than the reported prevalence of 0.074% in a Caucasian population in Northern Europe. This study included 1346 EOD patients who had neither a history of ketosis nor positive autoantibodies (islet cell autoantibodies, glutamic acid decarboxylase, and islet antigen 2 antibodies). We reported one patient with a rare homozygous pathogenic variant of the *WFS1* gene that lacked other phenotypes of WS, except for diabetes^6^. In another study from China that included 82 clinically diagnosed type 1 patients with negative autoantibodies (GADA, islet antigen 2 antibody, and ZnT8 antibody), normal BMI, and at least one episode of ketosis, 4 patients (4.9%) carried homozygous or compound heterozygous variants of *WFS1*. They were diagnosed with diabetes at ages ranging from 5 to 22 years and had no additional phenotypes of WS except for diabetes^5^. Similarly, in 152 clinically diagnosed MODY cases from South India, 3 (2.0%) cases with pathogenic or likely pathogenic WFS1 variations were identified^23^. In fact, the difference in age at the onset of diabetes and other phenotypes might be attributed to the genotype of the patients. In a systematic review of 412 patients with WS, de Heredia et al. reported that 94% of patients developed diabetes before 18 years of age^24^. Compared with these patients, the onset age of the patients with WFS1-DM (16–35 years) in this study was relatively higher, with 83% (5/6) of the patients diagnosed with diabetes after 18 years. Similar to previous studies, among the six patients with WFS1-DM identified in our study, only one (Patient 5) presented with deafness, while the other patients had isolated diabetes. Thus, the phenotypes of WFS1-related diabetes are sometimes atypical and often misdiagnosed as type 1 diabetes, type 2 diabetes, or MODY. Genetic testing is important for patients with early diagnosis and negative autoantibodies of type 1 diabetes to determine their real type of diabetes.

In this study, most patients with WFS1-DM carried one pathogenic allele and were non-ketotic and non-obese, consistent with a previously reported case^10^ of adult-onset diabetes due to a dominant WFS1 variant (p.Trp314Arg). In a study examining patients with WFS1-DM with type 1 diabetes, the patients with WFS1-DM had a lower incidence of diabetic ketosis, severe hypoglycemia, and microvascular complications^1,10,25,26^. This may be due to the long-term preservation of islet beta cell function in patients with WFS1-DM, in contrast to those with type 1 diabetes.

Our results show that almost all patients diagnosed with WFS1-DM in the current study presented with isolated diabetes, except for one patient with deafness. Of note, most of the patients with WFS1-DM in our study carried only one pathogenic allele, which may explain the later onset age compared to typical MODY and the lack of typical features of WS1. Unfortunately, we did not follow up these patients with WFS1-DM and did not know whether the patients will gradually develop WS-related signs in the future. No studies have been pubished reporting whether isolated diabetes caused by WFS1 mutations develops into the syndrome; however, further follow-up is necessary. In fact, previous studies have validated that autosomal dominant WFS1 variants can cause MODY and the neonatal diabetes phenotype^10^. Their diagnosis could be missed using the current strategy, which restricts the testing of syndromic genes in patients with characteristic clinical features. We recommend including *WFS1* in gene panel tests for MODY to enable the early diagnosis of atypical presentations and clinical benefits for diagnosed patients. To investigate the features of isolated diabetes (without OA, DI, or D) caused by *WFS1* variations, we reviewed the published literature and selected 51 patients from 13 studies (Supplementary Table 2). A total of 90% of patients were diagnosed before the age of 30 years. None of the patients were obese (BMI < 30 kg/m^2^). A total of 29% of these patients carried only one pathogenic allele, WFS1. The clinical phenotypes of patients with isolated diabetes caused by WFS1 variations are similar to those of our patients with WFS1-DM.

In this study, most patients with WFS1-DM required insulin treatment and lacked the clinical features of insulin resistance, and the serum C-peptide level in carriers with rare and predicted deleterious variants was lower than that in the EOD group without rare variants. These results indicate that insulin secretion is insufficient, and the function of islet beta cells is impaired, which is consistent with previous studies^10^. ER plays an important role in insulin biosynthesis and the folding of newly synthesized proinsulin in pancreatic beta cells. Mutant wolframin activates ER stress and unfolded protein response (UPR) in islet beta cells, causing impaired cell cycle progression and cell apoptosis^8^. In WFS1-deficient beta cells, the intensity of insulin secretory granules was reduced, and the acidification of secretory granules was impeded^27^. WFS1-deficient mice demonstrate impaired processes of conversion of proinsulin to insulin, resulting in an increased proinsulin-to-insulin ratio, which might partly explain our finding of decreased serum C-peptide levels in carriers of rare and predicted deleterious variants of the *WFS1* gene compared to those in non-carriers of rare *WFS1* variations.

Pioglitazone treatment protected beta cells from apoptosis and almost completely prevented diabetes development in Wfs1^−/−^ mice^28^, and GLP-1RAs were able to inhibit ER stress, improve β-cell function and reduce insulin dose in patients. Activation of the GLP-1 receptor signaling may alleviate insulin insufficiency in Wolfram syndrome and cellular stress caused by WFS1 deficiency^15,29^. In obesity and insulin resistance, hyperglycemia and dyslipidemia can cause ER stress, which leads to apoptosis and beta-cell dysfunction^30^. GLP1-receptor agonists (GLP1-RA) can improve insulin resistance and alleviate obesity^31^. Hence, GLP1-RA can indirectly alleviate ER stress. GLP1-mediated insulin secretion rate is significantly reduced in patients with common wfs1 variants^32^. After treatment with GLP1 agonists, ER stress was reduced in a rat model of WS, and pancreatic beta cell function, neuronal inflammation, sensorineural hearing loss, and optic atrophy were improved^33–35^. A case report^36^ described improvements in residual C-peptide secretion and neuroophthalmological disease progression in patients with WS after treatment with GLP1 agonists. Once these hypoglycemic agents have been validated as effective and safe in future clinical trials, mediating these interventions before individuals develop diabetes may be crucial. Therefore, clinical screening for timely genetic testing is necessary to identify patients with WFS1-DM.

In this study, we observed that the serum AST levels in patients with rare and predicted deleterious variants were higher than those without rare variants, which might be due to the difference in their susceptibility to liver injury between the two groups. The liver is an active organ involved in protein synthesis and processing, lipoprotein metabolism, and cholesterol biosynthesis. WFS1 is expressed in the liver tissues, and abnormal WFS1 activity may render the ER susceptible to stress in hepatocytes. ER stress accelerates liver damage in clinically ketotic cows^37^. Additionally, ER stress could trigger mitochondrial dysfunction via activation of the IRE1α^38^. Therefore, we speculate that once the function of wolframin is disrupted, ER stress is activated and aggravated in liver cells, which further leads to mitochondrial injury in liver cells and finally induces elevation in serum AST levels. However, further studies are needed to confirm this hypothesis.

There are some limitations to our study: (1) Pathogenicity evaluation was based on biological information; thus, some variations with uncertain significance identified in our study may be proven to be pathogenic in the future. This might have led to an underestimation of the prevalence of WFS1-DM, and functional experiments are needed in the future. Of note, ~ 5% of patients carried at least one rare predicted deleterious variant in this study. Although the effect of these variants was high enough to cause diabetes, it is possible to increase the susceptibility to diabetes, especially in the context of interactions with environmental risk factors. (2) Accurate detection of other major phenotypes of WS, such as a slight loss of hearing, was not recorded for the patients in this study. Our results indicated that hearing and vision were not severely affected in most patients with WFS1-DM in this cohort.

In summary, Chinese patients with WFS1-DM and EOD usually lack the typical clinical features of WS and are misdiagnosed with other types of diabetes. Patients with WFS1-DM are seldom obese and lack ketosis, and their clinical presentation is associated with insulin resistance. The function of islet beta cells in patients with EOD with rare and predicted deleterious WFS1 variants is severely impaired. Early identification and individualized treatment of patients with WFS1-DM are important for improving their clinical outcomes.

## Supplementary Information

Supplementary Information.

## Data Availability

All datasets are available from the corresponding authors upon reasonable request. The variation data of this study can be found in online repositories (https://www.ncbi.nlm.nih.gov/snp/), the submitted snp (ss) number(s) to each SNP can be found below: 2,137,544,442, 6,205,742,125, 6,205,742,126, 6,205,742,127, 6,205,742,128, 6,205,742,129, 6,205,742,130, 6,205,742,131, 6,205,742,132, 6,205,742,133, 6,205,742,134, 6,205,742,135, 6,205,742,136, 6,205,742,137, 6,205,742,138, 6,205,742,139, 6,205,742,140, 6,205,742,141, 6,205,742,142, 6,205,742,143, 6,205,742,144, 6,205,742,145, 6,205,742,146, 6,205,742,147, 6,205,742,148, 6,205,742,149, 6,205,742,150, 6,205,742,151, 6,205,742,152, 6,205,742,153, 6,205,742,154, 6,205,742,155, 6,205,742,156, 6,205,742,157, 6,205,742,158, 6,205,742,159, 6,205,742,160, 6,205,742,161.
