# Supplementary material for: The genetic and clinical characteristics of WFS1 related diabetes in Chinese early onset type 2 diabetes
Source: Sci Rep. 2023 Jun 5;13:9127. doi: 10.1038/s41598-023-36334-7 (PMC10241780; doi:10.1038/s41598-023-36334-7)
Supplement: Supplementary file 1 — Supplementary Information. [file 41598_2023_36334_MOESM1_ESM.docx]

**Supplementary Materials**

**
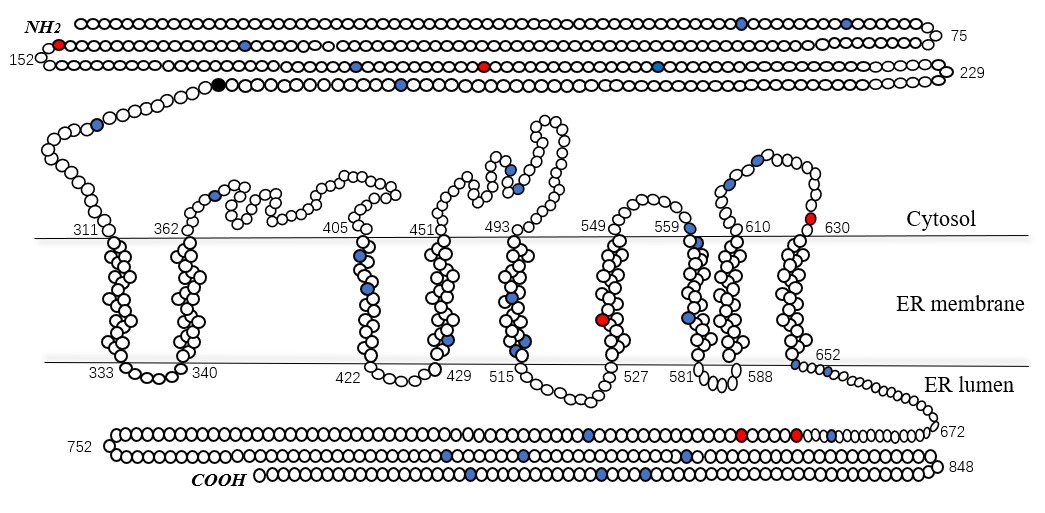
**

**Supplementary figure 1. the rare variations distribution in Wolframin**

blue solid circle = rare variation, red solid circle = variation detected in the patients with WFS1 related diabetes, black solid circle = p.lys287(the first amino acid encoded by exon 8), ER: endoplasmic reticulum


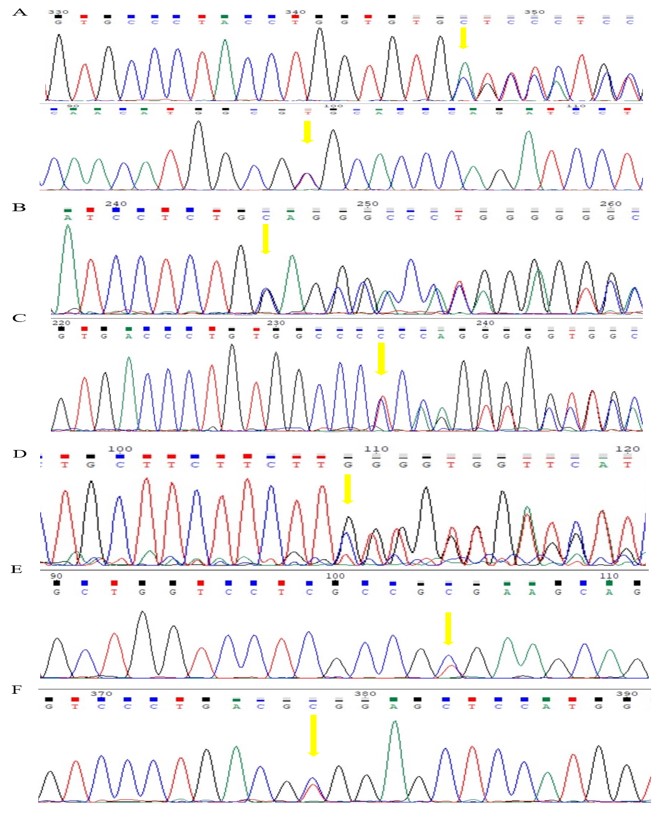


**Supplementary figure 2. DNA sequence analyses of WSLD patients**

A. the variations of Patient 1 (R685C and L535Lfs*3). B. the variation of Patient 2 (L689Lfs*18) C. the variation of Patient 3 (E694Gfs*16) D. the variation of Patient 4 (K193del) E. the variation of Patient 5 (A134V) F. the variation of Patient 6 (R629W)

Supplementary table 1. The classification of rare coding variants according to the guideline of the American College of Medical Genetics and Genomics.

| Position | Region | Codon change | AA change | ACMG |
| --- | --- | --- | --- | --- |
|  |  |  |  |  |
| 6279355 | Exon2 | GCG-GTG | A58V | US (PP4+PM1) |
| 6279382 | Exon2 | CAT-CTT | H67L | US (PP4+PM2+PM1+BP4) |
| 6290799 | Exon4 | GCG-GTG | A134V | LP (PP4+PM2+PP3+PM1) |
| 6290847 | Exon4 | GCG-GTG | A150V | US (PP4+PM1) |
| 6292998 | Exon5 | GCA-ACA | A179T | US (PP4+PP3 +PM1) |
| 6293030 | Exon5 | CAAG-C | K193del | LP (PP1+PP4+PP3+PM4+PM2+PP5) |
| 6293076 | Exon5 | GGC-AGC | G205S | US (PP4+PM1) |
| 6296873 | Exon7 | GAG-GCG | E273A | US (PP4+PP3+PM1) |
| 6302413 | Exon8 | ATG-ATA | M297I | US (PP4+PM2) |
| 6302626 | Exon8 | AGC-AGG | S368R | US (PP4+PP3 +PM2) |
| 6302741 | Exon8 | CAT-TAT | H407Y | US (PP4+PP2 +PM2) |
| 6302756 | Exon8 | GTC-CTC | V412L | US (PP4+PP3) |
| 6302816 | Exon8 | CTG-GTG | L432V | US (PP4+PP3+PM2) |
| 6302927 | Exon8 | TCG-ACG | S469T | US (PP4+PM2) |
| 6302937 | Exon8 | CCC-CTC | P472L | US (PP4+PM2) |
| 6303029 | Exon8 | GTC-ATC | V503I | US (PP4) |
| 6303030 | Exon8 | GTC-GGC | V503G | US (PP4+BP4) |
| 6303060 | Exon8 | TAT-TGT | Y513C | US (PP4+PP3+PM2) |
| 6303062 | Exon8 | CTC-TTC | L514F | US (PP4+PP3+PM2) |
| 6303126 | Exon8 | CTGGTGTGCTTCATG-C | L535Lfs*3 | P (PP4+PM2+PVS1) |
| 6303197 | Exon8 | GCC-ACC | A559T | US (PP4+PM1) |
| 6303203 | Exon8 | ATC-GTC | I561V | US (PP4+PM1) |
| 6303246 | Exon8 | GCC-GGC | A575G | US (PP4+PM2) |
| 6303368 | Exon8 | GCC-TCC | A616S | US (PP4+BP4) |
| 6303378 | Exon8 | TCT-TTT | S619F | US (PP4+PM2) |
| 6303407 | Exon8 | CGG-TGG | R629W | LP (PP4+PP3+PS3+PM2) |
| 6303479 | Exon8 | CGC-TGC | R653C | US (PP4+PM1) |
| 6303491 | Exon8 | ATG-TTG | M657L | US (PP4+PM1) |
| 6303575 | Exon8 | CGC-TGC | R685C | US (PP4+PM1+PM1) |
| 6303589 | Exon8 | TCTGCAGCCAC-T | L689Lfs*18 | P (PP4+PM2+PM1+PVS1) |
| 6303603 | Exon8 | GA-G | E694Gfs*16 | P (PP4+PM2+PM1+PVS1) |
| 6303644 | Exon8 | CGC-TGC | R708C | US (PP4+PM1) |
| 6303869 | Exon8 | TTC-CTC | F783L | US (PP4++PM1+BP4) |
| 6303891 | Exon8 | TCG-TTG | S790L | US (PP4+PM2+PM1) |
| 6303936 | Exon8 | CGG-CAG | R805Q | US (PP4+PM1) |
| 6304086 | Exon8 | TCA-TTA | S855L | US (PP4++PM1+BP4) |
| 6304097 | Exon8 | CGG-TGG | R859W | US (PP4+PM1) |
| 6304134 | Exon8 | GTG-GGG | V871G | US (PP4++PM1+PP3) |

RefSeq: NP_005996.2. AA change: amino acid change. P: pathogenic. LP: likely pathogenic. US: uncertain significance.

**Supplementary table 2. The systemic review on the clinical features of patients with isolated diabetes of WFS1-DM**

| Phenotype | Isolated diabetes  (n = 51) |
| --- | --- |
| Sex (M/F) | 14/14 |
| Age (years) | 16.0 (9.0,26.5) |
| Diagnosis age (years) | 10.0 (4.0,22.0) |
| Duration (years) | 7.0 (2.3,15.8) |
| BMI (kg/m^2^) | 23.53 ± 1.59 |
| Family history, n (%) | 27 (67.5) |
| Consanguinity, n (%) | 3 (16.7) |
| Heterozygote, n (%) | 11 (21.6) |
| Autoantibody, n (%) | 2 (0.0) |
| Ketosis, n (%) | 7 (35.0) |
| Diabetic retinopathy, n (%) | 3 (33.3) |
| Diabetic nephropathy, n (%) | 0 (0.0) |
| Insulin therapy/OHA/others | 42/3/2 |

Continuous variables in the table are expressed as medians (25^th^ to 75^th^ percentile) or means±standard deviations, and categorical variables are expressed as n (%).

BMI: body mass index, Autoantibody: including GADA (Glutamate Decarboxylase antibody). ICA (Insulin autoantibody), IA-2A (protein tyrosine phosphatase antibody). OHA: oral hypoglycemic agents.

References:

1. Zalloua, P.A., S.T. Azar, M. Delépine, N.J. Makhoul, H. Blanc, M. Sanyoura, A. Lavergne, K. Stankov, A. Lemainque, P. Baz, and C. Julier. WFS1 mutations are frequent monogenic causes of juvenile-onset diabetes mellitus in Lebanon. Human molecular genetics, 2008;17:4012-4021.

2. Li, M., S. Wang, K. Xu, Y. Chen, Q. Fu, Y. Gu, Y. Shi, M. Zhang, M. Sun, H. Chen, X. Han, Y. Li, Z. Tang, L. Cai, Z. Li, Y. Shi, T. Yang, and C. Polychronakos. High Prevalence of a Monogenic Cause in Han Chinese Diagnosed With Type 1 Diabetes, Partly Driven by Nonsyndromic Recessive Mutations. Diabetes, 2020;69:121-126.

3. Bansal, V., J. Gassenhuber, T. Phillips, G. Oliveira, R. Harbaugh, N. Villarasa, E.J. Topol, T. Seufferlein, and B.O. Boehm. Spectrum of mutations in monogenic diabetes genes identified from high-throughput DNA sequencing of 6888 individuals. BMC medicine, 2017;15:213.

4. Saint-Martin, C., D. Bouvet, M. Bastide, and C.B. Chantelot. Gene Panel Sequencing of Patients With Monogenic Diabetes Brings to Light Genes Typically Associated With Syndromic Presentations. Diabetes, 2021,

5. Bonnycastle, L.L., P.S. Chines, T. Hara, J.R. Huyghe, A.J. Swift, P. Heikinheimo, J. Mahadevan, S. Peltonen, H. Huopio, P. Nuutila, N. Narisu, R.L. Goldfeder, M.L. Stitzel, S. Lu, M. Boehnke, F. Urano, F.S. Collins, and M. Laakso. Autosomal dominant diabetes arising from a Wolfram syndrome 1 mutation. Diabetes, 2013;62:3943-3950.

6. Sobhani, M., M.A. Tabatabaiefar, S. Ghafouri-Fard, A. Rajab, A. Hojjat, A.-M. Kajbafzadeh, and M.R. Noori-Daloii. Clinical and genetic analysis of two wolfram syndrome families with high occurrence of wolfram syndrome and diabetes type II: a case report. BMC medical genetics, 2020;21:13.

7. Yaghootkar, H., F. Abbasi, N. Ghaemi, A. Rabbani, M.N. Wakeling, P. Eshraghi, S. Enayati, S. Vakili, S. Heidari, K. Patel, F. Sayarifard, S. Borhan-Dayani, T.J. McDonald, S. Ellard, A.T. Hattersley, M.M. Amoli, R. Vakili, and K. Colclough. Type 1 diabetes genetic risk score discriminates between monogenic and Type 1 diabetes in children diagnosed at the age of <5 years in the Iranian population. Diabetic medicine : a journal of the British Diabetic Association, 2019;36:1694-1702.

8. Bansal, V., B.O. Boehm, and A. Darvasi. Identification of a missense variant in the WFS1 gene that causes a mild form of Wolfram syndrome and is associated with risk for type 2 diabetes in Ashkenazi Jewish individuals. Diabetologia, 2018;61:2180-2188.

9. Astuti, D., A. Sabir, P. Fulton, M. Zatyka, D. Williams, C. Hardy, G. Milan, F. Favaretto, P. Yu-Wai-Man, J. Rohayem, M. López de Heredia, T. Hershey, L. Tranebjaerg, J.-H. Chen, A. Chaussenot, V. Nunes, B. Marshall, S. McAfferty, V. Tillmann, P. Maffei, V. Paquis-Flucklinger, T. Geberhiwot, W. Mlynarski, K. Parkinson, V. Picard, G.E. Bueno, R. Dias, A. Arnold, C. Richens, R. Paisey, F. Urano, R. Semple, R. Sinnott, and T.G. Barrett. Monogenic diabetes syndromes: Locus-specific databases for Alström, Wolfram, and Thiamine-responsive megaloblastic anemia. Human mutation, 2017;38:764-777.

10. Artuso, R., A. Provenzano, B. Mazzinghi, L. Giunti, V. Palazzo, E. Andreucci, A. Blasetti, R.M. Chiuri, F.E. Gianiorio, P. Mandich, M. Monami, E. Mannucci, and S. Giglio. Therapeutic implications of novel mutations of the RFX6 gene associated with early-onset diabetes. Pharmacogenomics J, 2015;15:49-54.

11. Yuca, S.A., N.D. Rendtorff, H. Boulahbel, M. Lodahl, L. Tranebjaerg, Y. Cesur, M. Dogan, C. Yilmaz, C. Akgun, and M. Acikgoz. Rapidly progressive renal disease as part of Wolfram syndrome in a large inbred Turkish family due to a novel WFS1 mutation (p.Leu511Pro). Eur J Med Genet, 2012;55:37-42.

12. Johansson, S., H. Irgens, K.K. Chudasama, J. Molnes, J. Aerts, F.S. Roque, I. Jonassen, S. Levy, K. Lima, P.M. Knappskog, G.I. Bell, A. Molven, and P.R. Njolstad. Exome sequencing and genetic testing for MODY. PLoS One, 2012;7:e38050.

13. Cano, A., C. Rouzier, S. Monnot, B. Chabrol, J. Conrath, P. Lecomte, B. Delobel, P. Boileau, R. Valero, V. Procaccio, V. Paquis-Flucklinger, S. French Group of Wolfram, and B. Vialettes. Identification of novel mutations in WFS1 and genotype-phenotype correlation in Wolfram syndrome. Am J Med Genet A, 2007;143A:1605-12.
